# Antibody responses after first and second Covid-19 vaccination in patients with chronic lymphocytic leukaemia

**DOI:** 10.1038/s41408-021-00528-x

**Published:** 2021-07-30

**Authors:** H. Parry, G. McIlroy, R. Bruton, M. Ali, C. Stephens, S. Damery, A. Otter, T. McSkeane, H. Rolfe, S. Faustini, N. Wall, P. Hillmen, G. Pratt, S. Paneesha, J. Zuo, A. Richter, P. Moss

**Affiliations:** 1 Institute of Immunology and Immunotherapy, University of Birmingham, Birmingham, B15 2TT UK; 2 Institute of Cancer and Genomic Sciences, University of Birmingham, Birmingham, B15 2TT UK; 3 Institute of Applied Health Research, University of Birmingham, Birmingham, B15 2TT UK; 4 National infection Service, Public Health England, Porton Down, Salisbury, SP4 OJG UK; 5 Cancer Research UK Clinical Trials Unit, University of Birmingham, B15 2TT Birmingham, UK; 6 St. James’s University Hospital, Leeds Teaching Hospitals NHS Trust, Leeds, LS9 7TF UK; 7 Queen Elizabeth Hospital, University Hospitals Birmingham, Birmingham, B15 2TH UK; 8 Birmingham Heartlands Hospital, University Hospitals Birmingham, Bordesley Green East, B9 5SS Birmingham, UK

**Keywords:** Translational research, Adaptive immunity, Chronic lymphocytic leukaemia

## Abstract

B-cell chronic lymphocytic leukaemia (CLL) is associated with immunosuppression and patients are at increased clinical risk following SARS-CoV-2 infection. Covid-19 vaccines offer the potential for protection against severe infection but relatively little is known regarding the profile of the antibody response following first or second vaccination. We studied spike-specific antibody responses following first and/or second Covid-19 vaccination in 299 patients with CLL compared with healthy donors. 286 patients underwent extended interval (10–12 week) vaccination. 154 patients received the BNT162b2 mRNA vaccine and 145 patients received ChAdOx1. Blood samples were taken either by venepuncture or as dried blood spots on filter paper. Spike-specific antibody responses were detectable in 34% of patients with CLL after one vaccine (*n* = 267) compared to 94% in healthy donors with antibody titres 104-fold lower in the patient group. Antibody responses increased to 75% after second vaccine (*n* = 55), compared to 100% in healthy donors, although titres remained lower. Multivariate analysis showed that current treatment with BTK inhibitors or IgA deficiency were independently associated with failure to generate an antibody response after the second vaccine. This work supports the need for optimisation of vaccination strategy in patients with CLL including the potential utility of booster vaccines.

## Introduction

Chronic lymphocytic leukaemia (CLL) is associated with profound immune dysregulation that progresses over the disease course. The underlying aetiology is multifactorial, with hypogammaglobulinaemia, impaired cellular immunity and therapy-related immunosuppression commonly observed [1]. These perturbations in immunity predispose patients to an increased risk of infection and infection-related mortality remains a common cause of death [2]. Vaccination against common infectious agents is of paramount importance in supportive care but vaccine-induced immune responses and associated clinical efficacy are often reduced in this patient group.

SARS-CoV-2 is a novel coronavirus and has led to a global pandemic with over 3.2 million deaths to date. Several studies have shown increased rates of morbidity and mortality after SARS-CoV-2 infection in patients with CLL and this is exacerbated by the age of many patients with this condition [3–5].

Novel vaccines against Covid-19 have shown remarkable efficacy and are likely to play a major role in control of the current pandemic [6]. BNT162b2 and ChAdOx1 utilise nucleoside-modified RNA or adenovirus-based platforms, respectively, and incorporate the SARS-CoV-2 spike protein as vaccine immunogen. Both vaccines are given as two doses with the BNT162b2 vaccine approved for a 3-week interval whilst clinical responses after ChAdOx1 are improved with a longer period between prime and boost [7]. However, several countries, including the UK, have elected to adopt an ‘extended interval’ vaccination regimen of between 10–12 weeks between BNT162b2 doses in order to maximise population coverage after a single vaccine.

Covid-19 vaccines offer the potential to provide patients with CLL with substantial clinical protection from SARS-CoV-2 infection but there is concern regarding the efficacy of vaccine responses in this group. Studies over many years have shown immune responses to vaccination are impaired in patients with B-CLL [8–10]. This is seen most particularly in the response to novel immunogens [8, 9]. Attenuated vaccine-induced immunity is seen across many stages of the disease course but immune function deteriorates most particularly in heavily treated patients. Vaccine responses are also suppressed in patients who are undergoing treatment with BTK inhibitors [8, 11]. There is relatively little information to date regarding the efficacy of Covid-19 vaccination in patients with CLL. Examination of 44 patients following 2 doses of mRNA vaccines recently reported a response rate of 52% [12]. Similarly, in a larger cohort of patients who had received the second BNT162b2 mRNA vaccine following a standard 3-week interval between doses, antibody responses were detected in 40%, with higher rates in patients with clinical remission after treatment, compared with a response rate of only 16% for patients on current treatment [13]. No current data exists studying ChAdOx1 in patients with CLL or for those on extended interval schedules.

Here we present an interim assessment of spike-specific antibody response in patients with CLL following BNT162b2 or ChAdOx1 vaccination. Due to usage of an ‘extended interval’ vaccine protocol within the UK for most patients to date, antibody responses have been assessed after single vaccination, with a smaller proportion assessed following the second dose. We show that immune responses are impaired in most patients and that IgA deficiency and current therapy with BTKi are independent risk factors for poor response.

## Methods

Patients with a diagnosis of CLL or small lymphocytic leukaemia (SLL) were recruited to the study. The work was performed under the CIA UPH IRAS approval (REC 20\NW\0240) and conducted according to the Declaration of Helsinki and good clinical practice. Informed consent was obtained in person or by remote consultation. Dates and subtype of SARS-CoV-2 vaccination were obtained together with self-reported information on disease stage and date of CLL diagnosis, CLL treatment and infection history. Infection history was considered ‘positive’ in cases of 2 or more serious or 3 or more respiratory infections in a 1-year period. Participants were asked about personal shielding and previous symptoms compatible with SARS-CoV-2 infection. Participant demographics can be found in Table 1.

**Table 1:** Details of patient cohort.

		Total cohort	DBS result		Serum result	
			First vaccine	Second vaccine	First vaccine	Second vaccine
**Number of patients**		299	267	55	86	12
**Age**	**Median (years)**	69	69	70	70	82.5
	**IQR**	63–74	63–74	65–75	65–76	80–85
	**Range**	43–96	43–96	48–87	49–96	52–87
**Sex**	**Men**	159 (53%)	143 (54%)	27 (49%)	47 (55%)	7 (58%)
	**Women**	140 (47%)	124 (46%)	28 (51%)	39 (45%)	5 (42%)
**Vaccine received**	**Pfizer**	154 (52%)	135 (51%)	38 (69%)	39 (45%)	12 (100%)
	**AstraZeneca**	145 (49%)	132 (49%)	17 (31%)	47 (55%)	0
**Time from vaccine to result**	**Median (days)**		43	18	47	39
	**IQR**		36 to 52	16 to 27	39 to 55	35 to 53
	**Range**		3 to 85	8 to 56	24 to 80	29 to 56
**Time since diagnosis**	**Median (months)**	79	81	75	87	220.5
	**IQR**	39–142	39–139	35–176	46–156	143–245
	**Range**	3–411	3–345	5–411	7–279	76–411
**Stage**	**A**	264 (88%)	233 (87%)	53 (96%)	78 (91%)	11 (92%)
	**B**	15 (5.0%)	14 (5.2%)	2 (3.6%)	1 (1.2%)	1 (8.3%)
	**C**	20 (6.7%)	20 (7.5%)	0	7 (8.1%)	0
**Previous treatment**	**W&W**	181 (61%)	157 (59%)	40 (73%)	55 (64%)	9 (75%)
	**1 line**	77 (26%)	73 (27%)	9 (16%)	23 (27%)	2 (17%)
	**2 lines**	28 (9.4%)	25 (9.4%)	4 (7.3%)	7 (8.1%)	1 (8.3%)
	**3+ lines**	13 (4.3%)	12 (4.5%)	2 (3.6%)	1 (1.2%)	0
**On BTKi**		60 (20%)	56 (21%)	8 (15%)	18 (21%)	0
**On venetoclax**		6 (2.0%)	6 (2.2%)	0	1 (1.2%)	0
**Previous chemotherapy**		71 (24%)	65 (24%)	8 (15%)	16 (19%)	2 (17%)
**Previous anti-CD20**		80 (27%)	75 (28%)	7 (13%)	18 (21%)	2 (17%)
**Treatment planned**		8 (2.7%)	6 (2.2%)	2 (3.6%)	1 (1.2%)	0
**History of infections**	**Frequent infections**	76 (25%)	67 (25%)	18 (33%)	23 (27%)	3 (25%)
	**Hospitalisations**	60 (20%)	53 (20%)	10 (18%)	14 (16%)	2 (17%)
**Prophylactic antibiotics**		55 (18%)	53 (20%)	3 (5.5%)	20 (23%)	0
**IVIG**		17 (5.7%)	17 (6.4%)	0	3 (3.5%)	0
**Immunoglobulin deficiency**	**Number**	117	85	54	62	12
	**IgG (<6** **g/L)**	43 (37%)	34 (40%)	19 (35%)	24 (39%)	2 (17%)
	**IgA (<0.8** **g/L)**	34 (29%)	27 (32%)	17 (32%)	17 (27%)	4 (33%)
	**IgM (<0.5** **g/L)**	56 (48%)	45 (53%)	16 (30%)	40 (65%)	5 (42%)

**Fig. 1:**
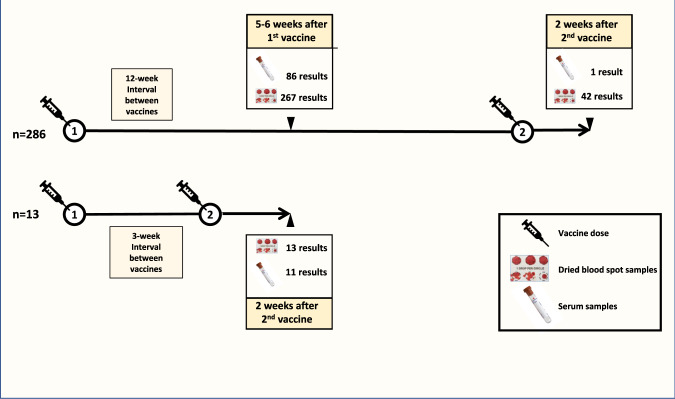
Infographic of study design and collection. Samples were collected from patients who had undergone dual vaccination with either an ‘extended interval’ (*n* = 286) or ‘standard interval’ regimen (*n* = 13). Antibody levels in serum samples obtained by phlebotomy or in eluates from dried blood spot samples were assessed at the indicated timepoints.

Samples were obtained following first or second vaccination. Local participants undertook a phlebotomy sample whilst those more distant donated a dried blood spot sample (DBS) from capillary blood after a finger prick (Fig. 1). A total of 93 healthy donor controls were recruited from local primary care networks.

### Roche Elecsys^®^ electrochemiluminescence immunoassay (ECLIA)

Serum was stored at −20 °C and defrosted prior to antibody analysis. IgG/A/M antibodies specific to SARS-CoV-2 were detected using electrochemiluminescence assays on the automated Roche cobas e801 analysers based at Public Health England (PHE) Porton. Calibration and quality control were performed as recommended by the manufacturer. Anti-nucleocapsid protein (NP) antibodies were detected using the qualitative Roche Elecsys^®^ AntiSARS-CoV-2 ECLIA (COV2, Product code: 09203079190), whilst anti-spike (S) antibodies were detected using the quantitative Roche Elecsys^®^ Anti-SARS-CoV-2 S ECLIA (COV2 S, Product code 09289275190). Anti-nucleocapsid results are expressed as a cut-off index (COI) value, with a COI value of ≥1.0 considered positive for anti-nucleocapsid antibodies. Anti-spike results are expressed as units per ml (U/ml), with samples with a result of ≥0.8 U/ml considered positive for anti-spike antibodies within the fully quantitative range of the assay: 0.4–2500 U/ml. Samples >2500 U/ml were diluted further (1:10, 1:100, and 1:1000) to within the quantitative range.

### Dried blood spot ELISA analysis

Dried blood spot analysis was carried out by Clinical Immunology Service (University of Birmingham). Capillary blood samples were collected on DBS cards (Ahlstrom Munksjo) from participants remotely and stored at room temperature. Samples were eluted in 250 microlitres of 0.05% phosphate-buffered saline-Tween 20 (PBS, Oxoid Tween-20, Sigma-Aldrich) per blood spot and incubated overnight (12–16 h) before centrifugation (10,600 x *g* for 10 min). The DBS eluate was then applied to a pre-coated 96-well ELISA plate (The Binding Site (TBS), Birmingham, UK) containing stabilised trimeric SARS-CoV-2 spike glycoprotein and detecting IgG, IgA and IgM antibody isotypes [14]. The performance characteristics for this assay were assessed in 162 non-hospitalised mild to moderate disease PCR-positive individuals and 707 presumed COVID-19 negative samples from pre-2019. Sensitivity was 96.3% (92.1−98.6) and specificity 99.3% (98.4−99.8). The ELISA output result was reported as a ratio relative to a monoclonal spike-specific calibration antibody standard and multiplied by the previously determined cut-off co-efficient to maintain batch-to-batch consistency, defined as 1.31. A positive result was classed as a ratio of 1 or more.

### Serum immunoglobulin measurement

Quantification of IgG, IgA and IgM was evaluated using the COBAS 6000 (Roche) at the University of Birmingham Clinical Immunology Service. For analysis of DBS eluates, levels were multiplied by 10.7 to account for dilution factor and following validation of paired DBS and healthy serum samples.

### Statistical analysis

Data were tested for normality using Kolmogorov–Smirnov analysis. For comparative analysis of antibody titres between healthy donors and patients with CLL, Mann–Whitney U-tests were performed. Spearman rank correlation was used for comparing assay platforms and for correlating total serum Ig and time since diagnosis against anti-S responses. For comparison of anti-S response by CLL treatment groups, Kruskal–Wallis was performed with post-hoc Dunn’s analysis and paired 1^st^ and 2^nd^ vaccine responses, Wilcoxon’s matched-pairs signed rank test was used. Binary logistic regression of clinical variables was performed to test for associations with positive antibody response after the second vaccine. All analysis was performed using Graphpad prism v9.1.0 for Mac (San Diego, California USA) aside from logistic regression for which SPSS Statistics v27.0 for Windows (Armonk, NY: IBM Corp.).

## Results

### Patient characteristics

A total of 299 patients were enrolled in the study together with 93 age-matched healthy donors. The median age of the patient group was 69 years (IQR 63-74) and 159 of the CLL patients (53%) were male. A total of 154 patients had received the Pfizer-BioNTech mRNA vaccine (BNT162b2) whilst 145 received the AstraZeneca/Oxford ChAdOx1 adenovirus vaccine.

286 patients (96%) were vaccinated with an ‘extended interval’ regimen of 10–12 weeks between the first and second vaccine. In this group blood samples were taken after the first vaccine in most cases in order to assess the immune response to single vaccine delivery. Thirteen patients (4%) were vaccinated according to the ‘standard interval’ for the mRNA vaccine of a 3-week time period between first and second dose. In this group samples were taken only after the second vaccine. Matched samples after first and second vaccine were available in 27 cases. The median time to sample collection following the first vaccination was 43 days (IQR: 36–52 days; *n* = 267) whilst the median time to sample collection following second vaccination was 18 days (IQR: 14–28; *n* = 55) (Fig. 1).

One hundred and eighty one patients (61%) were at stage A and were untreated with ‘watch and wait’ monitoring. One hundred and eighteen (39%) had received treatment for CLL, of which 66 (22%) were actively being treated. The type and number of lines of therapy is shown in Table 1. Seventy-six patients (25%) reported a clinical history of frequent infections whilst 60 (20%) also reported previous hospital admission for infection. Fifty-five patients were on prophylactic antibiotics (18%) and 17 were on immunoglobulin replacement therapy (6%). The median time from diagnosis to sample collection was 79 months (IQR 39–142 months).

### Antibody responses following first vaccination are markedly reduced in patients with CLL

Antibody responses in patients undergoing extended-interval vaccination were determined at 5–6 weeks after the initial vaccine. Analysis was performed on venepuncture serum samples (S1) in 86 patients and a simultaneous dried blood spot sample (DBS1) was also taken at the same time in 82 subjects. An additional 185 patients provided a DBS1 sample following the first vaccine such that a total of 267 samples were available for analysis.

**Fig. 2:**
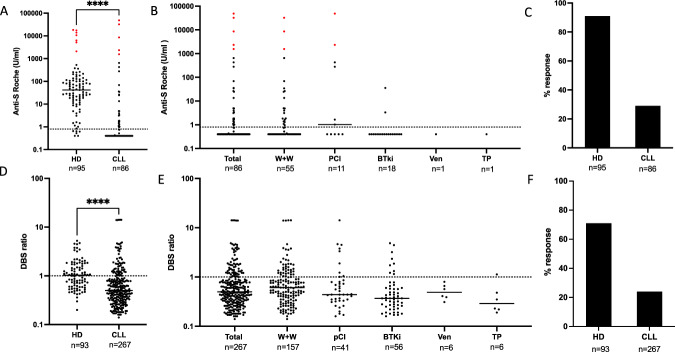
Antibody responses in patients with CLL following first Covid-19 vaccine. **A** Antibody responses to SARS-COV19 Spike following first vaccine in sera from healthy donors (HD) and patients with CLL, as measured by Roche assay. Red indicates those previously exposed (PE) to SARS-CoV-2 (median HD 41.6 vs CLL 0.4 (104 fold change)); cut off for positivity at 0.8 shown by dotted line. **B** Antibody responses to SARS-CoV-2 Spike in patients with CLL following first vaccine, by management stage, as measured by Roche (Total patients; Watch and Wait (W+W); Previous Chemo-immunotherapy but not on active therapy (pCI); Bruton Tyrosine Kinase therapy (BTKi); Venetoclax therapy (Ven); Treatment planned (TP). **C** Bar chart to show the percentage response after first vaccine in healthy donors and CLL measured by Roche. **D** Antibody responses to SARS-CoV-2 Spike from DBS in healthy donors (HD) and patients with CLL, as measured by The Binding Site (TBS) assay using DBS eluates (median 1 vs 0.5; *p* < 0.0001; (cut off positivity shown at 1). **E** Antibody responses to SARS-CoV-2 Spike in patients with CLL following first vaccine, by management stage, as measured by TBS using DBS eluates (Kruskal–Wallis *p* = 0.0054; post hoc Dunn’s analysis *p* < 0.0056 W+W vs BTKi). **F** Bar chart to show the percentage response after first vaccine in HD and CLL measured by TBS using DBS eluates.

Twenty-nine of the 86 serum S1 samples (34%) gave a positive anti-spike antibody response using the Roche platform. This overall response rate was 2.8-fold lower than that of age-matched healthy donors where 94% were found to have detectable antibodies. Median antibody titres were also markedly reduced and were 104-fold lower in patients compared to healthy donors (0.4 vs 41.6 U/ml respectively; *p* < 0.0001) (Fig. 2A, C). The median titre amongst patients with a positive result was 3.96 (10.5-fold lower than healthy donors) whilst the median titre response of those who responded to the first vaccine but had no evidence of previous infection was 2.49 (16-fold lower than healthy donors).

Several studies have now shown that people who have had a previous natural SARS-CoV-2 infection exhibit particularly strong immune responses following Covid-19 vaccination. Previous infection can be determined by the presence of nucleocapsid-specific antibodies and these were detected in 5 donors with CLL. Within this group, median antibody titres within S1 samples were boosted by a remarkable 21,450-fold to reach a median value of 8580 U/ml (naturally infected median 8580 U/ml vs no previous infection 0.4 U/ml; *p* < 0.0001). Of note these values are comparable to the levels of 10,700 U/ml in samples from healthy donors with previous infection after one vaccine (*p* > 0.999).

Given the clinical heterogeneity amongst patients with CLL we next assessed antibody responses in relation to clinical status and management. Patients were divided into 5 groups: ‘watch and wait’; previously completed chemo-immunotherapy (median 68 months (IQR 35-115) since last treatment); current treatment with BTK inhibitor; current treatment with venetoclax; and ‘plans to start treatment imminently’. Analysis of serum samples showed that responses were low in all five groups and, although numbers within the subgroups were small, patient responses were particularly suppressed amongst those on current therapy or those who were due to start therapy in the near future (Fig. 2B).

Following analysis of the serum samples we went on to determine the spike-specific antibody response from dried blood spot samples (DBS) using a spike-specific ELISA (The Binding Site (TBS)). These DBS1 samples were available in 267 patients and compared to values from 93 age-matched healthy donors following single vaccination. Analysis of paired samples showed a strong correlation between the spike-specific antibody response detected by serum and DBS within the patient group (*r* = 0.65; *p* < 0.0001) (Supplementary Fig. 1A) and confirms previous work within healthy donors [15].

Antibody responses were detectable in 63 of the 267 DBS1 patient samples (24%) which compares to the value of 34% from the serum analysis. Positive responses were detectable in 71% of the control group and as such were somewhat lower than responses obtained from analysis of serum samples (Fig. 2D, F). The magnitude of antibody response from DBS eluates was also lower within the patient group compared to controls (median ratio to calibrator of 0.5 vs 1; *p* < 0.0001) (Fig. 2D). Amongst the 24% of patients where a positive antibody response was seen, a similar median ratio was observed in patients with CLL and healthy donors (CLL: 1.76 vs HD 1.71). Analysis of DBS1 eluate samples showed low responses across all patient groups. Comparison between subgroups showed that patients on BTKi therapy were less likely to develop a positive antibody response compared to those on watch and wait (*p* = 0.0056) (Fig. 2E). No difference was observed between patients who received the BNT162b2 and ChAdOx1 vaccines (Supplementary Fig. 1B).

### Antibody responses improve after second vaccination but remain low compared to age-matched controls

**Fig. 3:**
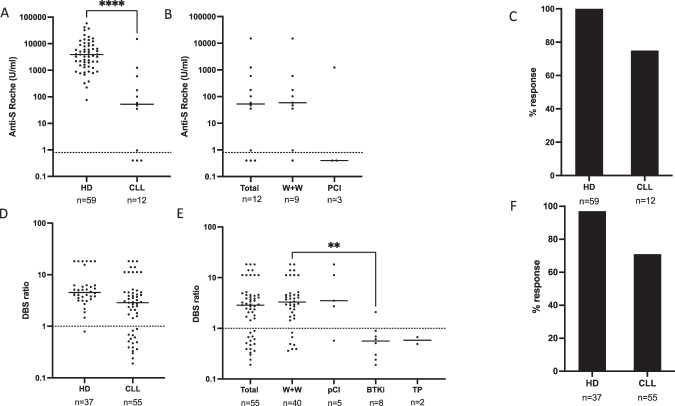
Antibody responses in patients with CLL following second Covid-19 vaccine. **A** Antibody responses to SARS-CoV-2 Spike following second vaccine in sera from healthy donors (HD) and patients with CLL, as measured by Roche assay. No donors had evidence of previous exposure (cut off for positivity at 0.8 indicated by dotted line). **B** Dot plot of antibody responses to SARS-CoV-2 Spike in patient with CLL following second vaccine, by management stage is shown, (W+W watch and wait; PCi previous chemo-immunotherapy but not on active therapy) as measured by Roche. **C** Bar chart to show the percentage response after second vaccine in HD and CLL measured by Roche. **D** Antibody responses to SARS-CoV-2 spike following second vaccine in healthy donors (HD) and patients with CLL, as measured by TBS assay (cut off for positivity indicated by the dotted line at a ratio of 1). **E** Antibody responses to SARS-CoV-2 Spike in patient with CLL following second vaccine, by management stage, DBS testing and analysed by TBS assay (Kruskal–Wallis *p* < 0.0045 and Dunn’s analysis for BTKi therapy and W+W *p* = 0.03). Watch and Wait (W+W); Previous Chemo-immunotherapy but not on active therapy (pCI); Bruton Tyrosine Kinase therapy (BTKi); Treatment planned (TP). **F** Bar chart to show the percentage response after second vaccine in HD and CLL measured by TBS assay using DBS eluates.

We next went on to assess antibody responses at 2–3 weeks following the second vaccine. At the current time, due to widespread adoption of the ‘extended interval’ vaccine regimen in the UK, samples are available on only 12 serum and 55 DBS samples (termed S2 and DBS2 samples, respectively). Spike-specific antibody responses were identified in 9 of the 12 serum samples from the patient group (75%) compared to a 100% response rate in healthy age-matched controls (*n* = 59) (Fig. 3C). The titre of this anti-spike antibody response was 74-fold lower in patients with CLL compared to healthy age-matched donors without previous infection (*n* = 59) (53 U/ml vs 3900 U/ml; *p* < 0.0001) (Fig. 3A, C). Amongst the 75% of patients with a positive antibody response, the median response was 102 U/ml. Eight of these patients were on ‘watch and wait’ management (Fig. 3B) and no patients in the group had serological evidence of previous natural SARS-CoV-2 infection.

**Fig. 4:**
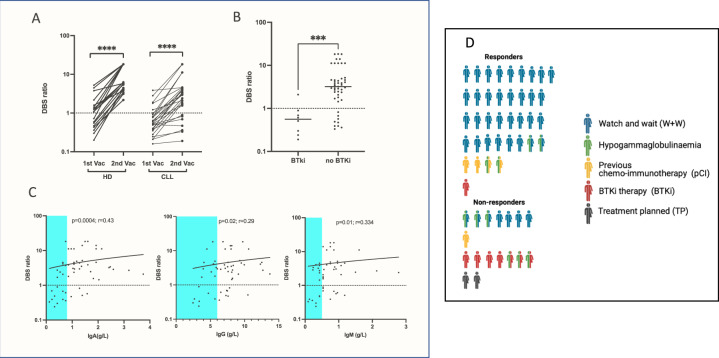
Determinants of Covid-19 vaccine response in patients with CLL. **A** Paired analysis of antibody responses to SARS-CoV-2 Spike in healthy donors (HD) and patients with CLL after first and second vaccine (Analysis by TBS ELISA, HD median post 1^st^ and 2^nd^ vaccine 1.0 & 5.8 vs CLL median 0.5 & 3.0, respectively) with cut off for positivity shown by dotted line ratio = 1). **B** Dot plot showing antibody responses to second vaccine in patients with CLL taking a Bruton Tyrosine Kinase inhibitor (BTKi) and those not on BTKi (median BTKi 0.6 vs No BTKi 3.2 *p* = 0.0002) as measured by TBS assay. **C** Correlation between antibody response to SARS-CoV-2 following second vaccine and serum immunoglobulin. Antibody levels were measured by TBS ELISA and shown against total serum IgA (*p* = 0.0004; *r* = 0.43, IgA deficiency (0.8 g/L) is highlighted in blue); total serum IgG (*p* = 0.02; *r* = 0.29, IgG deficiency (6 g/L) highlighted in blue) and total serum IgM (*p* = 0.01, *r* = 0.334, IgM deficiency (<0.5 g/L) highlighted in blue). **D** Infographic of patients with CLL according to the presence or absence of antibody response following double vaccination according to disease characteristics (*n* = 55).

Spike-specific antibody responses were then assessed in the 55 DBS2 samples. Antibodies were detectable in 71% of patients (39/55) compared to 97% (36/37) of samples from healthy donors (*n* = 37) (Fig. 3F). Antibody levels, as assessed by the DBS2 ratio, were also significantly lower amongst the 55 patients (2.9 vs 4.5 healthy donors; (*p* = 0.0004; Fig. 3D), whilst those with a detectable antibody response had a median ratio of 3.86. Analysis of responses in relation to clinical status showed lower antibody levels in patients on BTKi therapy (Figs. 3E and 4B).

Paired samples after the first and second vaccine were available for 27 donors and revealed similar fold-increment in antibody levels between timepoints within both the patient group and 19 age-matched healthy donors (Fig. 4A). As such these data indicate that patients with CLL get an equivalent proportionate antibody response after the second vaccine although values both before and after this vaccine remain lower than those in the control group.

Total serum immunoglobulin levels were also determined on DBS2 samples for subsequent assessment of their relationship to spike-specific antibody response. Seventeen of the 55 patients (35%) were found to be IgG deficient, 19 (32%) were IgA deficient and 16 (30%) were IgM deficient (Fig. 4C). A combined deficiency of IgG and IgA was present in 10 (18%) patients (Fig. 4D).

### Determinants of response to second vaccine

The relative importance of individual clinical and laboratory variables on the probability of developing a positive spike-specific antibody response after second vaccination were then assessed in univariate analysis against values obtained from DBS2 samples (Table 2).

**Table 2:** Univariate analysis of determinants of positive antibody response after second vaccine.

Variable	Odds ratio (95% CI)	*P* value
**Age (increasing)**	0.99. (0.93 to 1.05)	0.73
**Duration of CLL (increasing)**	1.0. (0.99 to 1.002)	0.15
**lgA (normal)**	**10.7 (2.7 to 43)**	**0.001**
**lgG (normal)**	2.5. (0.73 to 8.8)	0.14
**lgM (normal)**	2.9 (0.83 to 10.3)	0.095
**BTKI treatment (Yes)**	**0.034 (0.004 to 0.31)**	**0.003**
**Infection history (Yes)**	0.35 (0.1 to 1.2)	0.086

The age of the patient and the duration of time since diagnosis were not associated with the probability of generating a positive antibody response (Supplementary Fig. 2). Antibody response rates were lower in patients who reported a history of severe infection but this did not reach statistical significance. Serum concentrations of IgG, IgA or IgM all showed positive correlations with antibody response but this remained significant only for IgA in multivariate analysis (IgA: OR 9.1; 2–42, *p* = 0.005). Current therapy with BTK inhibitors was associated with markedly reduced likelihood of a positive response to vaccination and remained significant in multivariate analysis (OR: 0.05; 95% CI: 0.004–0.58, *p* = 0.016).

## Discussion

SARS-CoV-2 vaccines have proven highly effective in protection against severe Covid-19 but there remains considerable concern about their efficacy in patients with immune suppression [16]. Here we show that immune responses elicited after the first and second vaccine are substantially reduced in patients with chronic lymphocytic leukaemia. These findings raise a number of questions in relation to optimisation of vaccine protection in this vulnerable cohort.

The median age of our cohort was 69 years and as such it would be expected that immune senescence will play a role in vaccine response [17]. Importantly we were able to compare the patient responses with a large age-matched control group. Antibody responses within the patient group after one vaccination were low and detectable in only 34% compared to 94% of age-matched controls. The antibody titre was also over 100-fold lower at this time. These results are comparable with the 18% response rate in a haematological cancer cohort that included 11 CLL patients [16].

Interestingly, 5 of the 86 donors for which serological samples were available showed the presence of nucleocapsid-specific antibodies indicating previous natural infection and in this group the antibody response after first vaccine was remarkably high and directly comparable to those seen in previously infected healthy donors after vaccination. Indeed, antibody levels were markedly higher than those seen in previously uninfected patients after two Covid-19 vaccines. As such, immune memory after natural infection appears able to overcome the impaired antibody response to the initial vaccine in the patient group. Similar findings in relation to the ‘vaccine-priming’ effect of previous natural infection have previously been reported [15, 18, 19]. Of interest, only 2 of these 5 patients reported symptoms compatible with SARS-CoV-2 infection over the last 12 months suggesting that asymptomatic infection occurs amongst patients with CLL and that mortality rates may have therefore been overestimated [3, 5]. One limitation to this finding may be that some patients with CLL who had previous natural infection may lack a nucleocapsid-specific antibody response in the convalescent serum due to antibody waning, secondary immunodeficiency or hypogammaglobulinaemia.

Encouragingly, antibody responses increased after the second vaccine and were positive in 75% of serum samples and 71% of DBS eluates at this time point. These values are somewhat higher than the response rate of 40% in a recent study of 167 patients after the second BNT162b2 vaccine [13]. Further assessment of donors within our study at this time point is ongoing as more samples are collected. Of note, most patients in this study were studied after extended interval vaccination and this may potentially serve to boost antibody responses following the second vaccine [7]. Our study has found no difference in antibody levels following the BNT162b2 or ChAdOx1 vaccines to date.

Of interest, spike-specific antibody levels within patients after second vaccination were broadly comparable to those seen in the control group after a single vaccine which is noteworthy given the clinical protection demonstrated following a single vaccine within the general population [20].

We were further interested to see how vaccine responses varied in relation to clinical and laboratory features of individual patients. Patient age, or time since original diagnosis, did not impact significantly on vaccine response. In contrast the serum level of immunoglobulin was a notable determinant, with higher levels of all three isotypes associated with improved antibody response rate in univariate analysis. IgA deficiency emerged as the most significant predictor of poor vaccine response and normal levels were associated with a 10-fold increase in the probability of a positive response after second vaccination. IgA is the first of the immunoglobulin classes to diminish in patients with CLL [21] and this is reflected in this cohort where 32% of participants were deficient in IgA. IgA levels at diagnosis have also been shown to predict infection risk [22] and IgA deficiency has been associated with poor responses to pneumococcal polysaccharide vaccines [23]. Herishanu et al. also found that having normal immunoglobulins predicted better response rates to the BNT162b2 vaccination [13].

Bruton tyrosine kinase inhibitor therapy was a strong and independent predictor of negative antibody response after the second vaccine. BTKi therapy has transformed the management of patients with CLL over the past decade but its impact on vaccine response has been previously reported and is unsurprising given the pivotal role of BTK in B-cell activation [8, 11]. It would appear advisable that patients who are being considered for BTKi therapy should receive their Covid-19 vaccination prior to therapy where possible. However, we also observed suboptimal vaccine responses in untreated patients who were planning to start therapy in the near future and this is likely to reflect the immunosuppressive impact of active disease.

In contrast, positive antibody responses were observed in 83% of patients who were on ‘watch and wait’ management and this was particularly true for those with normal serum immunoglobulin levels.

Relatively few patients in our cohort were in remission from previous chemotherapy but it appears that antibody responses in this group are also more robust and this would be compatible with previously published work [13]. Other limitations of our work include patient self-reporting for infection history and previous COVID-19 infection, a lack of nucleocapsid-specific antibody assessment in the DBS samples, inability to link to current haematological parameters, and the relatively small number of samples after the second vaccine as a result of the extended interval programme.

Our findings raise questions regarding optimal vaccination policy for patients with CLL and the potential need for additional management. The great majority of the UK population is receiving the Covid-19 vaccine with an ‘extended interval’ of 10–12 weeks between doses. As such, in this interim report the majority of samples were obtained after the first vaccine but before the second dose and suggest that patients with CLL should be considered for early delivery of the second vaccine. However, antibody responses may remain suboptimal even after two vaccines. One option might be to consider a third ‘booster’ vaccine and this is being implemented in some countries for patients in other risk groups. At this stage it is not clear if a third vaccine will indeed act to further boost antibody responses although the substantial increment after the second boost and the high antibody titres observed following natural infection suggest that this may be possible. It is possible that vaccination may serve to provide sufficient ‘immune-priming’ to protect against severe disease from subsequent infection even in the absence of a measurable spike-specific antibody response. Indeed, we have not yet assessed the profile of spike-specific cellular immunity following vaccination and, although cellular immune responses are also typically suboptimal in patients with CLL, these are likely to provide some protection from severe disease [24]. However, antibody levels are emerging as a correlate of immune protection [20, 25] and as such the administration of prophylactic SARS-CoV-2-specific antibodies to patients at the greatest clinical risk could be valuable.

In conclusion we show that antibody responses following Covid-19 vaccination are reduced in patients with CLL, with patients who are IgA-deficient or on BTKi therapy at particular risk for failure to develop a response. It is now critical that the clinical efficacy of vaccination is determined in this patient group using data linkage from large population datasets and this assessment is underway in many countries. This information, together with assessment of immune correlates, will be important to guide the ongoing requirement for behavioural adjustments such as social distancing.

## Supplementary information

Supplementary figure 1

Supplementary figure 2

AJ check list
