# Supplementary material for: Antibody responses after first and second Covid-19 vaccination in patients with chronic lymphocytic leukaemia
Source: Blood Cancer J. 2021 Jul 30;11(7):136. doi: 10.1038/s41408-021-00528-x (PMC8323747; doi:10.1038/s41408-021-00528-x)
Supplement: Supplementary file 1 — Supplementary figure 1 [file 41408_2021_528_MOESM1_ESM.pptx]

## Slide 1
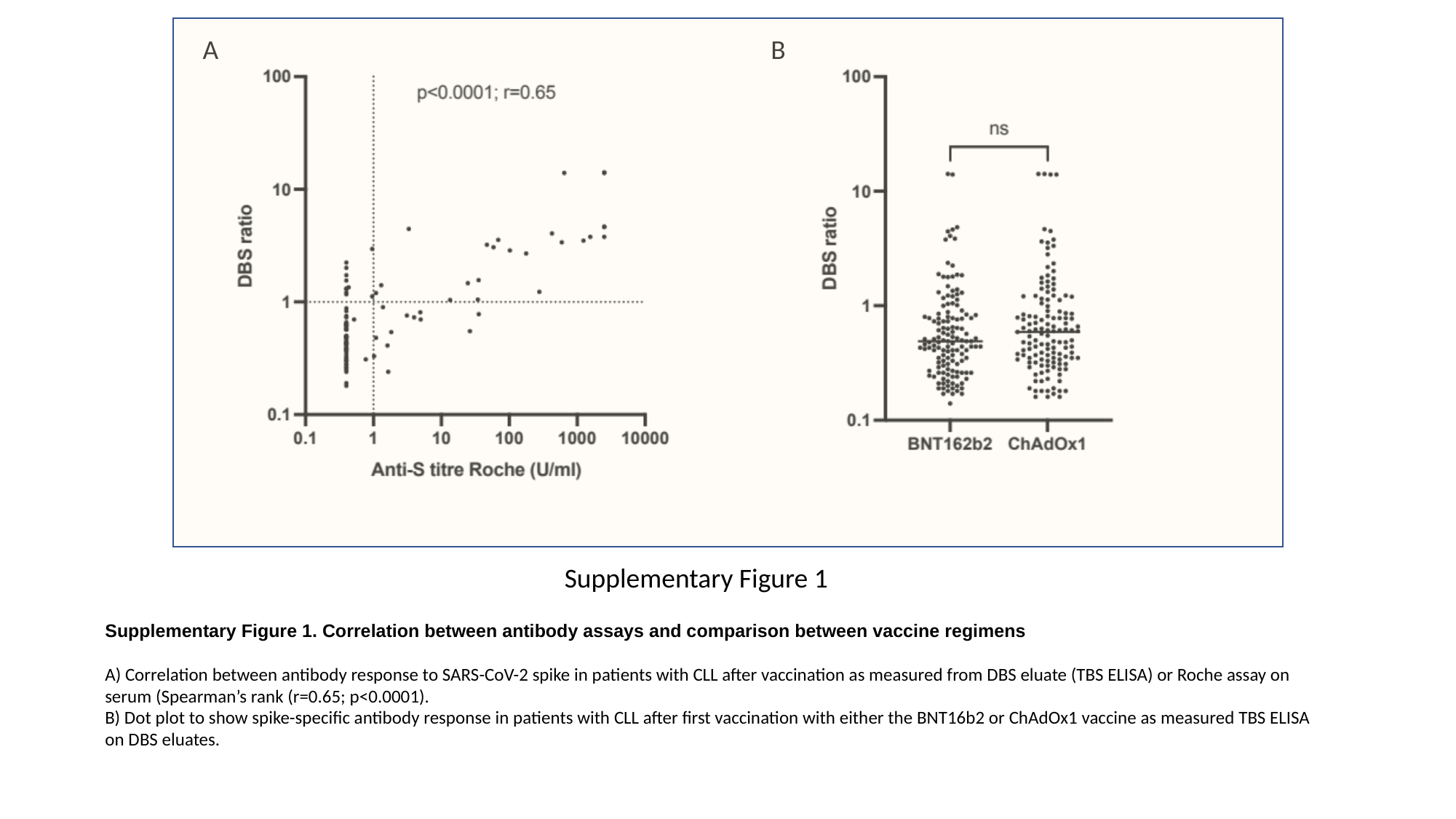

A
B
Supplementary Figure 1
Supplementary Figure 1. Correlation between antibody assays and comparison between vaccine regimens
A) Correlation between antibody response to SARS-CoV-2 spike in patients with CLL after vaccination as measured from DBS eluate (TBS ELISA) or Roche assay on serum (Spearman’s rank (r=0.65; p<0.0001).
B) Dot plot to show spike-specific antibody response in patients with CLL after first vaccination with either the BNT16b2 or ChAdOx1 vaccine as measured TBS ELISA on DBS eluates.
