# Supplementary material for: Antibody responses after first and second Covid-19 vaccination in patients with chronic lymphocytic leukaemia
Source: Blood Cancer J. 2021 Jul 30;11(7):136. doi: 10.1038/s41408-021-00528-x (PMC8323747; doi:10.1038/s41408-021-00528-x)
Supplement: Supplementary file 2 — Supplementary figure 2 [file 41408_2021_528_MOESM2_ESM.pptx]

## Slide 1
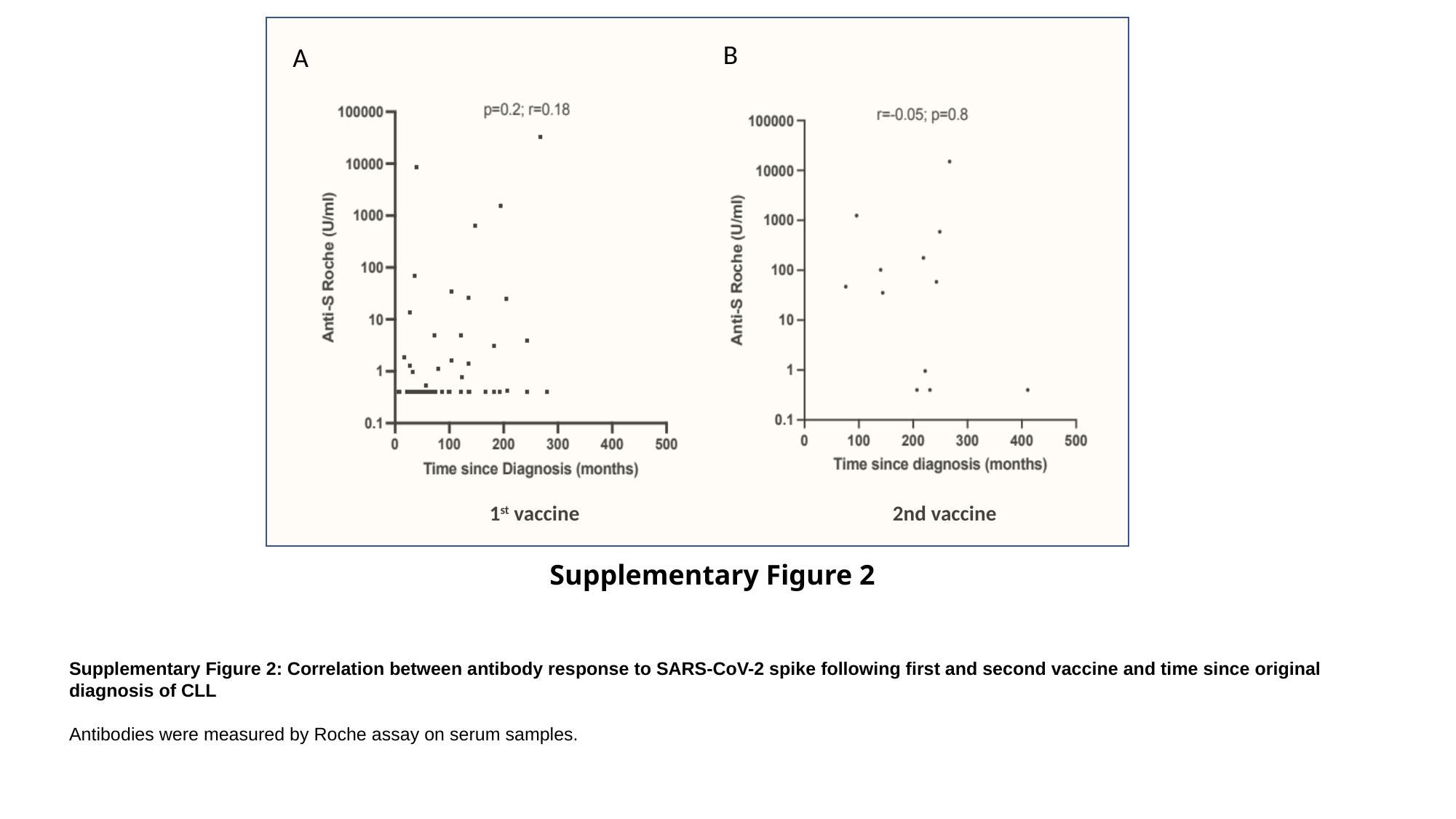

B
A
1st vaccine
2nd vaccine
Supplementary Figure 2
Supplementary Figure 2: Correlation between antibody response to SARS-CoV-2 spike following first and second vaccine and time since original diagnosis of CLL
Antibodies were measured by Roche assay on serum samples.
